# Incidence of hearing loss following COVID-19 among young adults in South Korea: a nationwide cohort study

**DOI:** 10.1016/j.eclinm.2024.102759

**Published:** 2024-07-29

**Authors:** Hye Jun Kim, Seogsong Jeong, Kyuwoong Kim, Joon Don Lee, Yun Hwan Oh, Michelle J. Suh

**Affiliations:** a Department of Biomedical Sciences, Seoul National University College of Medicine, Seoul, South Korea; b Department of Biomedical Informatics, Korea University College of Medicine, Seoul, South Korea; c National Cancer Control Institute, National Cancer Center, Goyang, Republic of Korea; d Graduate School of Cancer Science and Policy, National Cancer Center, Goyang, Republic of Korea; e Department of Otorhinolaryngology, Jeju National University Hospital, Jeju National University College of Medicine, Jeju, South Korea; f Department of Family Medicine, Chung-Ang University Gwangmyeong Hospital, Chung-Ang University College of Medicine, Gwangmyeong-si, South Korea

**Keywords:** Hearing loss, Severe acute respiratory syndrome coronavirus 2, Cohort study, Inverse probability of treatment weighting, Young adults

## Abstract

**Background:**

The association of COVID-19 with hearing loss (HL) is unclear among young adults and needs to be investigated. This study was conducted to determine the association of COVID-19 with HL and sudden sensorineural hearing loss (SSNHL) in young adults.

**Methods:**

This nationwide population-based cohort study used data from the Korea Disease Control and Prevention Agency-COVID-19-National Health Insurance Service. The study population consisted of young adult citizens aged 20–39 years without a history of HL. All participants were followed up from July 1, 2022 until HL, death, or December 31, 2022. A positive diagnosis of SARS-CoV-2 infection was determined through laboratory testing employing real-time reverse transcription polymerase chain reaction assays using nasopharyngeal or oropharyngeal swabs. The primary and secondary outcomes were HL and SSNHL, respectively. Age, sex, household income, Charlson comorbidity index, COVID-19 vaccination, hypertension, diabetes, and dyslipidemia-adjusted subdistribution hazard ratios (aSHRs) and 95% confidence intervals (CIs) were evaluated using the Fine–Gray subdistribution hazard regression model, considering overall death as a competing event to compare the aSHRs between COVID-19 positive and negative groups.

**Findings:**

A total of 6,716,879 young adults were eligible for the analyses. During 40,260,757 person-months (PMs) of follow–up, 38,269 cases of HL and 5908 cases of SSNHL were identified. The risk of HL (incidence: 11.9 versus 3.4/10,000 PMs; SHR, 3.51; 95% CI, 3.39–3.63; aSHR, 3.44; 95% CI, 3.33–3.56; *P* < 0.0001) and SSNHL (incidence: 1.8 versus 0.5/10,000 PMs; SHR, 3.58; 95% CI, 3.29–3.90; aSHR, 3.52; 95% CI, 3.23–3.83; *P* < 0.0001) was higher in COVID-19 group as compared to no COVID-19 group. In the sensitivity analyses that evaluated HL and SSNHL risks after adopting multiple imputations, utilizing inverse probability of treatment weighting, limiting study population to the cohort with a health screening examination, the results were consistent to the primary analysis.

**Interpretation:**

Our findings suggest a heightened risk of HL and SSNHL following COVID-19 in young adults. Due to study limitations, including the lack of objective audiological data, issues with generalizability to other populations, and the retrospective design, careful interpretation is necessary. Further studies with objective audiological data and a longer follow-up period are warranted.

**Funding:**

10.13039/501100008126IITP (Institute for Information & Communications Technology Planning & Evaluation; IITP-2024- RS-00156439) and Jeju National University Hospital Research Fund (2023).

**Research in context**

**Evidence before this study**

We searched PubMed for studies published up to January, 2024 using the terms "COVID-19" OR "SARS-CoV-2" AND "hearing loss" OR "sudden sensorineural hearing loss" AND "young adults". Several case reports and small-scale studies had suggested a potential link between COVID-19 and hearing impairments. However, large-scale population-based studies investigating the association between COVID-19 and hearing loss, particularly in young adults, were not available. Available evidence was focused on older populations or had small sample sizes without sufficient evidence on the association of COVID-19 with hearing loss in younger age groups.

**Added value of this study**

This nationwide population-based cohort study of 6,716,879 young adults aged 20–39 years in South Korea provides real-world evidence on the association between COVID-19 and both hearing loss (HL) and sudden sensorineural hearing loss (SSNHL) in this age group. Our findings demonstrate a significantly higher risk of HL and SSNHL in young adults with COVID-19 compared to those without, with adjusted sub-distribution hazard ratios of 3.44 and 3.52, respectively. The large sample size, nationwide database, and robust statistical analyses, including sensitivity analyses, provide evidence for this association that was previously understudied in young adults.

**Implications of all the available evidence**

The results of this study, combined with previous evidence, highlight a potentially underrecognized complication of COVID-19 in young adults. These findings suggest that healthcare providers should be aware of the increased risk of hearing impairments in young adult COVID-19 patients and consider appropriate screening and follow-up. The study also emphasize the need for further research into the biological mechanisms by which SARS-CoV-2 might affect auditory function, as well as potential therapeutic strategies. Additionally, these results may inform public health policies regarding COVID-19 management and vaccination strategies, emphasizing the importance of protecting young adults from infection to prevent potential long-term auditory complications. Future studies should focus on long-term follow-up of COVID-19 patients to assess the progression of hearing impairments, as well as investigating potential medical interventions to mitigate this risk.

## Introduction

To date, global cases of Coronavirus disease 2019 (COVID-19) cases have surpassed 770 million, resulting in almost 7 million reported deaths.^1^ COVID-19 is known to impact various body systems, including the respiratory, gastrointestinal, cardiovascular, and auditory systems.^2^^,^^3^

During the early stages of the pandemic, a researcher in Thailand reported a case involving an elderly woman who experienced both COVID-19 and sensorineural hearing loss (HL). The researcher suggested that the involvement of the brainstem in COVID-19 might be associated with neuroauditory issues.^4^ Similarly, a study conducted in Turkey reported that one out of every five patients presenting sudden sensorineural hearing loss (SSNHL) at an outpatient clinic had a concurrent diagnosis of COVID-19.^5^ This suggests that HL could be a nonspecific symptom of COVID-19. Degen et al.^6^ reported a case of sensorineural HL and suggested its potential link to bacterial or viral meningitis, where inflammation of the meninges triggered by a virus may extend to the cochlea, resulting in sudden-onset HL. In addition, viral infections have been implicated in causing direct or indirect damage to the inner ear.^7^^,^^8^ An analysis from a single institution reveals an increased incidence of SSNHL during the COVID-19 pandemic compared to the corresponding time frame in the previous year.^9^ A Danish cohort study involving 225 patients with COVID-19 found a significant proportion of patients experiencing HL.^10^ Despite efforts to determine the association of COVID-19 with the risk of HL or SSNHL, a systematic review concluded that whether COVID-19 contributes to a higher risk of HL or SSNHL remains unknown, possibly due to the limited scale of studies, such as case series.^11^

Recent case reports have documented sudden HL in young adults with no prior hearing issues after COVID-19 infection,^11^^,^^12^ suggesting that hearing problems among young individuals have emerged as a new public health issue following the COVID-19 pandemic. HL in young individuals can significantly impact their quality of life, academic and occupational performance, and social functioning.^13^ Moreover, young adults generally have fewer comorbidities and better overall health status compared to older individuals, with age-related hearing impairment being less pronounced.^14^

Despite conflicting results in case series and limited cohort studies examining the association between COVID-19 and the risk of HL, it is crucial to determine this association in a large-scale cohort controlled for confounding factors. In our study, we evaluated the risk of hearing HL and SSNHL following the COVID-19 outbreak in a nationwide cohort of young adults.

## Methods

### Study population

This cohort study utilized data from the Korea Disease Control and Prevention Agency (KDCA)-COVID-19-National Health Insurance Service (NHIS) cohort, by merging information from the KDCA COVID-19 registry with the NHIS database. The COVID-19 registry includes details such as the date of COVID-19 diagnosis, vaccination types, doses, and vaccination dates. The NHIS with approximately 97% of all Korean citizens offers comprehensive insurance coverage and collects diverse data, sociodemographic details, health screening examination results, medication prescription records, hospitalization, outpatient visits, disease diagnoses, and medical treatment records.^15^, ^16^, ^17^ The study period ranged from January 1, 2020 to December 31, 2022. From the KDCA-COVID-19-NHIS database, young adults aged 20–39 years in 2022 who had died or had a history of HL before the follow-up investigation were excluded. This study received approval from the Institutional Review Board of Jeju National Hospital (IRB number: JEJUNUH 2023-05-022). The need for informed consent was waived as the NHIS database adheres to strict confidentiality guidelines and anonymizes the data. This study was conducted in accordance with the STROBE guidelines.

### Exposures

The exposure variable was confirmed SARS-CoV-2 infection determined through laboratory testing employing real-time reverse transcription polymerase chain reaction assays using nasopharyngeal or oropharyngeal swabs. This diagnosis occurred before the initial follow-up investigation on July 1, 2022.

### Outcomes

The primary outcome, composite HL, was defined using the International Classification of Diseases, 10th revision, codes H90 and H91. The secondary outcome was the diagnosis of SSNHL based on the International Classification of Diseases, 10th revision, code H912. For the evaluation of HL risk and person-years, patients were followed up until the date of HL diagnosis, death, or December 2022. For the evaluation of SSNHL risk and person-years, patients were followed up until the date of SSNHL diagnosis, death, or December 2022; two analyses (HL and SSNHL) were independently carried out. HL was diagnosed when an individual hears sounds only at or above 25 dB (based on the average pure-tone hearing threshold of 0.5, 1, 2, and 4 kHz). The diagnostic criteria for SSNHL involved a rapid progression of the disease within 72 h with a HL of 30 dB or more at three consecutive frequencies in pure-tone audiometry.

### Covariates

For the primary cohort analyses, the adjustment variables included age (continuous; year), sex (categorical; male and female), household income (categorical; upper half and lower half), Charlson comorbidity index (CCI; categorical; 0, 1, and ≥2), and COVID-19 vaccination (categorical; none, single dose only, and completion of the primary series). For the analysis of the cohort with a health screening examination, additional adjustments were made for body mass index (continuous; kg/m^2^), systolic blood pressure (continuous; mmHg), fasting serum glucose (continuous; mg/dL), smoking (categorical; never, past, and current), alcohol consumption (categorical; yes and no), and moderate-to-vigorous physical activity (categorical; 0, 1–2, 3–4, and ≥5 time/week).

### Statistical analysis

All continuous variables, presented as mean (standard deviation [SD]), exhibited a normal distribution, while categorical variables were expressed as numerical values (%). Fine–Gray subdistribution hazard regression model, incorporating overall death as a competing event, estimated the subdistribution hazard ratios (SHRs) and 95% confidence intervals (CIs) for the risk of HL and SSNHL among young adults with COVID-19 compared to those without COVID-19. Non-COVID-19 participants were censored at the date of COVID-19 diagnosis. The incidences of HL and SSNHL were calculated per 10,000 person-months (PMs). The minimally adjusted model included age and sex as adjustment variables, while the fully adjusted model further incorporated household income, CCI, COVID-19 vaccination, hypertension, diabetes, and dyslipidemia.

Several sensitivity analyses were conducted using the Fine–Gray subdistribution hazard regression model. First, the multiple imputations of missing information for health screening examination results were adopted to evaluate the association of COVID-19 with the risk of HL or SSNHL. Second, we employed inverse probability of treatment weighting (IPTW) to account for potential selection bias in the KDCA-COVID-19-NHIS database. Young adults diagnosed with COVID-19 were assigned with a weight of one divided by their estimated propensity score computed from multiple logistic regression (i.e., the probability of being diagnosed with COVID-19 given the baseline characteristics of age, sex, household income, CCI, hypertension, diabetes, dyslipidemia, and COVID-19 vaccination). Conversely, young adults who were not diagnosed with COVID-19 were assigned with a weight of one divided by one minus their propensity score. After the implementation of IPTW, the pseudo-population where COVID-19 diagnosis assignment is random, mitigated the potential influence from confounding variables. For the third sensitivity analyses, we further excluded those who did not participate in the health-screening examination (n = 3,380,533) and those with missing information for the covariates (n = 3620) to construct a subpopulation who underwent a health screening examination to further adjust for health-screening examination results (Supplementary Figure S1).

Stratified analyses were performed to identify variables with a significant interaction on the risk of HL and SSNHL in the presence of COVID-19. Statistical significance was determined by a two–sided *P* < 0.05. SAS version 9.4 (SAS Institute, Cary, NC, USA) was used for all data mining, collection, and analyses.

### Role of the funding source

The funder of the study had no role in study design, data collection, data analysis, data interpretation, or writing of the report.

## Results

**Fig. 1:**
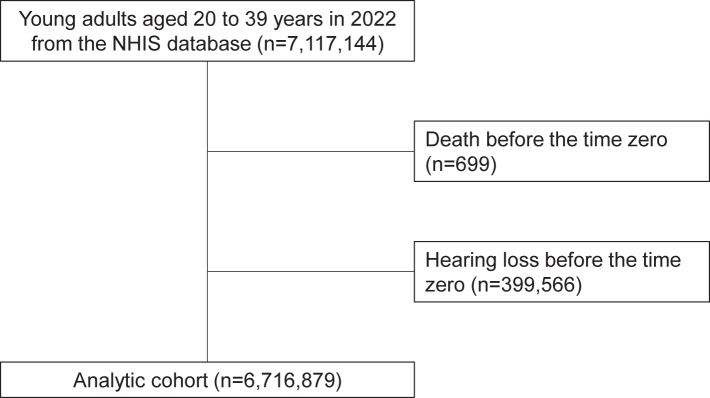
**Flow diagram for the inclusion of the young adults in South Korea**.

This retrospective cohort study identified 7,117,144 young adults aged 20–39 years in 2022 from the KDCA-COVID-19-NHIS database. After excluding individuals who died before July 1, 2022 (n = 699) and those with a history of HL before the follow-up investigation (n = 399,566), the remaining 6,716,879 young adults were included in the analytic cohort (Fig. 1). Table 1 displays the baseline characteristics of 6,716,879 young adults in the main analytic cohort. The mean age was 29.6 (SD 5.7) years, and 49.0% (n = 3,291,387) were male. A total of 904,542 (13.5%) young adults had a Charlson comorbidity index (CCI) ≥2. At baseline, 4,834,766 (72.0%) young adults had COVID-19 and 6,253,050 (93.1%) had completed the primary series of COVID-19 vaccinations.

**Table 1:** Descriptive characteristics of the young adults in South Korea.

	Young adults (n = 6,716,879)
Age, years, mean (SD)	29.6 (5.7)
Sex, n (%)	
Male	3,291,387 (49.0)
Female	3,425,492 (51.0)
Household income, n (%)	
Upper half	3,410,283 (50.8)
Lower half	3,306,596 (49.2)
CCI, n (%)	
0	2,407,795 (35.8)
1	3,404,542 (50.7)
≥2	904,542 (13.5)
Hypertension, n (%)	181,678 (2.7)
Diabetes, n (%)	59,765 (0.9)
Dyslipidemia, n (%)	167,997 (2.5)
COVID-19, n (%)	
Yes	4,834,766 (72.0)
No	1,882,113 (28.0)
COVID-19 vaccination, n (%)	
None	390,046 (5.8)
Single dose	73,783 (1.1)
Completion of primary series	6,253,050 (93.1)

Acronyms: SD, standard deviation; CCI, Charlson comorbidity index; COVID-19, coronavirus disease 2019.

During 40,260,757 PMs of follow–up, 38,269 cases of HL were identified. Table 2 presents the association of COVID-19 with the risk of incident HL and SSNHL. The incidence of HL was 11.9/10,000 PMs in the COVID-19 group, which was over 3-fold higher than the non-COVID-19 group. Similarly, the incidence of SSNHL was over 3-fold higher in the COVID-19 group compared to the non-COVID-19 group. In the fully adjusted model, risks of HL (adjusted subdistribution hazard ratio [aSHR], 3.44; 95% CI, 3.33–3.56; *P* < 0.0001) and SSNHL (aSHR, 3.52; 95% CI, 3.23–3.83; *P* < 0.0001) were significantly higher in COVID-19 group compared to the non-COVID-19 group.

**Table 2:** Subdistribution hazard ratios for the association of COVID-19 with the risk of hearing loss and sudden sensorineural hearing loss.

	No COVID-19 (n = 1,882,113)	COVID-19 (n = 4,834,766)	*P* value
Hearing loss			
Event	3820	34,449	
PMs	11,279,982	28,980,775	
Incidence/10,000 PMs	3.4	11.9	
SHR (95% CI)	1.00 (reference)	3.51 (3.39–3.63)	<0.0001
aSHR (95% CI)^a^	1.00 (reference)	3.49 (3.38–3.61)	<0.0001
aSHR (95% CI)^b^	1.00 (reference)	3.44 (3.33–3.56)	<0.0001
SSNHL			
Event	579	5329	
PMs	11,316,491	29,069,429	
Incidence/10,000 PMs	0.5	1.8	
SHR (95% CI)	1.00 (reference)	3.58 (3.29–3.90)	<0.0001
aSHR (95% CI)^a^	1.00 (reference)	3.56 (3.26–3.87)	<0.0001
aSHR (95% CI)^b^	1.00 (reference)	3.52 (3.23–3.83)	<0.0001

SHR calculated using the Fine–Gray subdistribution hazard regression model.

Acronyms: COVID-19, coronavirus disease 2019; PM, person-month; SHR, subdistribution hazard ratio; CI, confidence interval; aSHR, adjusted subdistribution hazard ratio; SSNHL, sudden sensorineural hearing loss.

a Adjusted for age and sex.

b Adjusted for age, sex, household income, Charlson comorbidity index, COVID-19 vaccination, hypertension, diabetes, and dyslipidemia.

The first sensitivity analysis, which adopted multiple imputations for the missing variables from health screening examination (Supplementary Table S1), showed that the risks of HL and SSNHL in the COVID-19 group were similar to the primary analysis (Supplementary Table S2). In the second sensitivity analysis following IPTW (Supplementary Table S3), the incidences of HL and SSNHL remained consistent across the COVID-19 and non-COVID-19 groups compared to the incidence before IPTW. Moreover, the risks of HL (aSHR, 3.44; 95% CI, 3.38–3.51; *P* < 0.0001) and SSNHL (aSHR, 3.52; 95% CI, 3.36–3.70; *P* < 0.0001) were significantly higher in the COVID-19 group compared to the non-COVID-19 group (Supplementary Table S4). A total of 3,336,346 young adults underwent a health screening examination, and 3,332,726 young adults had complete information for all health screening-related variables. The descriptive characteristics of the cohort with a health screening examination, consisting of non-COVID-19 and COVID-19 participants are presented in Supplementary Table S5. The incidence of HL and SSNHL was higher in the cohort with a health screening examination than in the main analytic cohort. Consistent with the results of the primary analysis, the cohort with a health screening examination revealed that COVID-19 is associated with higher risks of HL (aSHR, 3.55; 95% CI, 3.39–3.72; *P* < 0.0001) and SSNHL (aSHR, 3.43; 95% CI, 3.06–3.85; *P* < 0.0001; Supplementary Table S6).

**Fig. 2:**
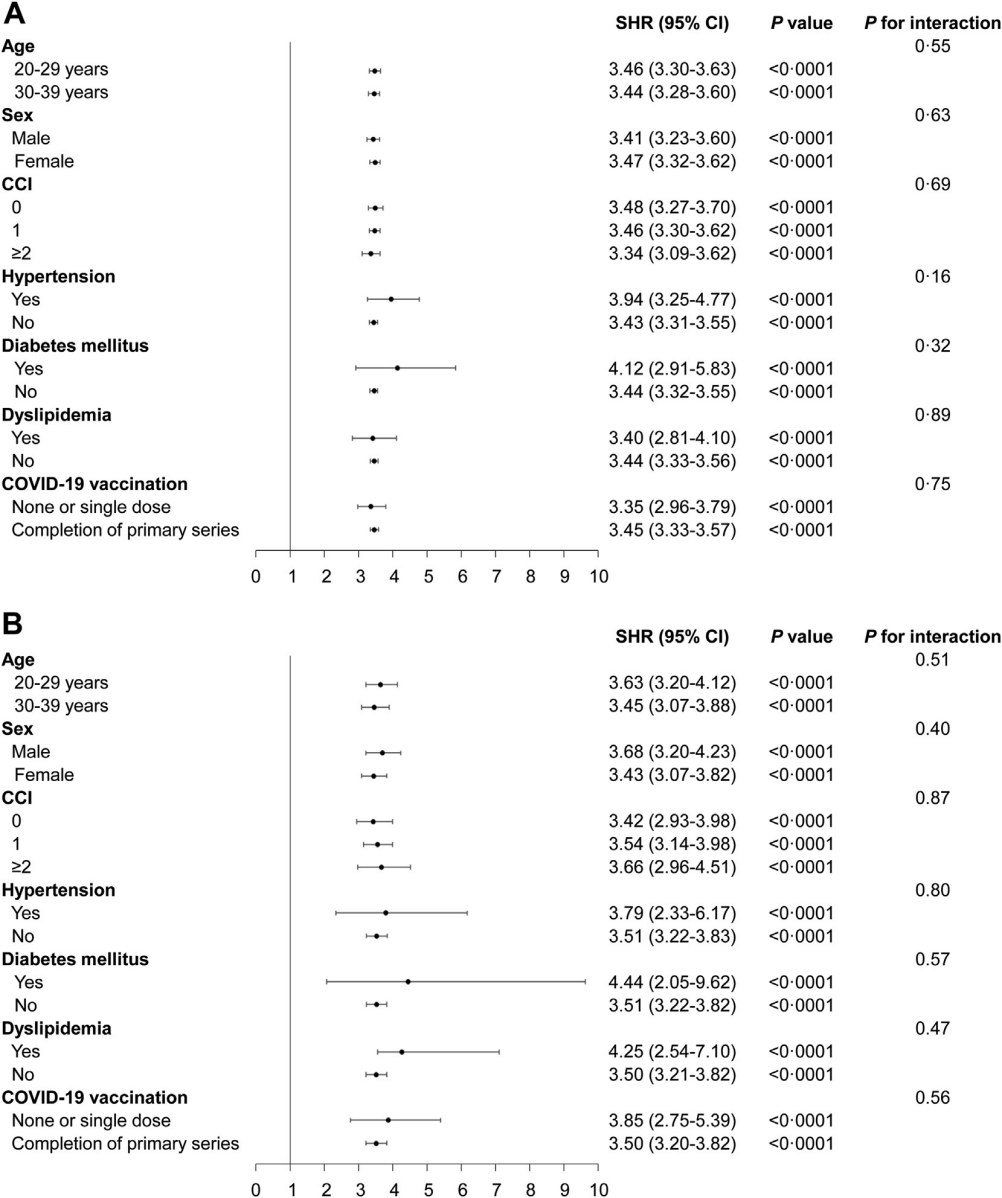
**Subgroup analyses on association of COVID-19 with the risk of hearing loss and sudden sensorineural hearing loss among young adults.** SHRs (95% CIs) calculated using the Fine–Gray subdistribution hazard regression model after adjustments for age, sex, household income, Charlson comorbidity index, COVID-19 vaccination, hypertension, diabetes, and dyslipidemia. (A) SHRs for the risk of hearing loss for patients with COVID-19. (B) SHRs for the risk of sudden sensorineural hearing loss for patients with COVID-19. Acronyms: COVID-19, coronavirus disease 2019; SHRs, subdistribution hazard ratios; CI, confidence interval.

In the stratified analyses, the highest risk of HL associated with COVID-19 was observed in young adults with diabetes mellitus (aSHR, 4.12; 95% CI, 2.91–5.83; *P* < 0.0001; Fig. 2A). No variable exhibited a significant interaction affecting the risk of HL associated with COVID-19. Similarly, the highest risk of SSNHL linked to COVID-19 occurred in young adults with diabetes mellitus (aSHR, 4.44; 95% CI, 2.05–9.62; *P* < 0.0001), followed by those with dyslipidemia (aSHR, 4.25; 95% CI, 2.54–7.10; *P* < 0.0001; Fig. 2B). No variable showed a significant interaction influencing the risk of developing SSNHL due to COVID-19. In addition, no significant interaction was found for COVID-19 vaccination in evaluating the association of COVID-19 and the risk of HL or SSNHL when participants were stratified according to their COVID-19 vaccination status (Table 3).

**Table 3:** Subdistribution hazard ratios for the association of COVID-19 with the risk of hearing loss and sudden sensorineural hearing loss according to the COVID-19 vaccination.

	No COVID-19 (n = 1,882,113)	COVID-19 (n = 4,834,766)	*P* value	*P* for interaction
Hearing loss				0.80
None	1.00 (reference)	3.36 (2.94–3.85)	<0.0001	
Single dose	1.00 (reference)	3.29 (2.41–4.48)	<0.0001	
Completion of primary series	1.00 (reference)	3.45 (3.33–3.57)	<0.0001	
SSNHL				0.74
None	1.00 (reference)	3.48 (2.44–4.96)	<0.0001	
Single dose	1.00 (reference)	7.84 (2.48–24.83)	<0.0001	
Completion of primary series	1.00 (reference)	3.50 (3.20–3.82)	<0.0001	

Data are subdistribution hazard ratio (95% confidence interval) calculated using the Fine–Gray subdistribution hazard regression model after adjustments for for age, sex, household income, Charlson comorbidity index, hypertension, diabetes, and dyslipidemia.

Acronyms: COVID-19, coronavirus disease 2019; SSNHL, sudden sensorineural hearing loss.

## Discussion

This nationwide population-based cohort study initially identified higher risk of incident HL and SSNHL among young adults in the COVID-19 group. After IPTW, the COVID-19 groups showed 3.44-fold and 3.52-fold higher risks of HL and SSNHL, respectively, compared to the non-COVID-19 group. These associations remained significant after adjusting for metabolic profiles and lifestyle behaviors.

A systematic review encompassing 28 case reports, case series, and cross-sectional studies estimated a 7.6% prevalence of HL since the onset of COVID-19 pandemic.^18^ When comparing the number of cases before and during the pandemic, studies showed mixed results, with some observing a decrease or similar incidence of cases and others noting an increase.^18^ Particularly within the young adult population, several case reports have documented COVID-19-related HL and SSNHL with no prior health issues. Pokharel S. et al.^19^ reported the case of a 27-year-old healthy man who experienced acute onset of unilateral SSNHL one month after a COVID-19 diagnosis. Another study by Gerstacker K. et al.^20^ detailed a case of a 38-year-old man with normal hearing prior to infection, who developed sudden bilateral HL after treatment in the intensive care unit for severe COVID-19 symptoms. Lang B. et al.^21^ also presented a case of a 30-year-old healthy female hospital nurse who developed unilateral SSNHL and tinnitus after COVID-19 diagnosis, with subsequent symptom resolution. Despite these studies suggesting a potential association in young adult patients with COVID-19 and an increased risk of HL or SSNHL, these findings are limited to descriptive studies, limiting our ability to fully elucidate the true association between COVID-19 and subsequent HL and SSNHL in this population.

Several hypotheses have been proposed to explain the risk of COVID-19-related HL and SSNHL; however, the evidence remains limited. Firstly, the inner ear's susceptibility to viruses may lead to direct damage caused by SARS-CoV-2 infection.^22^ Viral infections, including those from the Herpesviridae family, Paramyxoviridae family, Lassa virus, hepatitis viruses, and influenza viruses, are known to cause neurological symptoms such as smell disorders, facial paralysis, or SSNHL.^5^^,^^23^ Damage to the cochlear and perilymphatic tissues induced by viral activity,^24^ as observed in conditions like cochlear nerve neuritis, cochleitis, and the stress response of inner ear antigen, might contribute to the development of these manifestations.^5^ Additionally, an animal study using mice induced with labyrinthitis by the herpes simplex virus has demonstrated HL following viral infection.^25^ The causes are attributed to the infection in epithelial cells within the stria vascularis and subsequent apoptosis of infected cells, as well as apoptosis of many uninfected cells in the organ of Corti.^25^ A previous study identified the presence of SARS-CoV-2 in the middle ear for nearly a month following infection with the Omicron variant, indicating the virus's capacity to persist for extended periods and potentially contribute to the onset of HL.^26^ The receptor-binding domain of the SARS-CoV-2 spike protein binds to the human angiotensin-converting enzyme 2 (ACE2) receptor.^27^ This receptor plays a critical role in the renin-angiotensin system, thereby initiating the transmission of the virus.^27^ Notably, ACE2 receptors have been detected not only in the respiratory tract but also in extrapulmonary tissues, including the organ of Corti, stria vascularis, middle ear, and eustachian tube, in both animal and human models.^23^^,^^28^ In addition to ACE2, both transmembrane protease serine 2 (TMPRSS2) and Furin contribute to the potent infectivity of SARS-CoV-2 within the human body, and they have also been found in the middle ear spaces, eustachian tube, and the cochlea of mice.^28^ Indeed, inflammation in the inner ear, such as cochlear hair cell dysfunction, has been observed in patients with SARS-CoV-2-driven HL.^24^^,^^29^ The presence of ACE2 receptors, TMPRSS2, and Furin in otolaryngological tissues may explain their susceptibility to SARS-CoV-2 infection and subsequent hearing impairment.

Furthermore, it is hypothesized that microvascular damage, resulting from inadequate arterial supply to the cochlea, micro bleeding in cerebral tissue and the labyrinth following viral infection,^22^^,^^30^^,^^31^ an immunologic response in the cochlea,^7^^,^^32^ and the generation of proinflammatory cytokines and reactive oxygen species within the inner ear triggering an abnormal stress response,^33^ could serve as potential mechanisms. However, further biological and genetic investigations are required to confirm the mechanisms underlying COVID-19-induced HL and SSNHL in young adults.

The present study has several limitations that should be considered. Firstly, there is a possibility of selection bias, as individuals who sought COVID-19 diagnosis might have been more likely to seek HL diagnosis. Also, our study relied on PCR-confirmed COVID-19 cases, which may not account for untested or asymptomatic individuals. However, recent studies have shown that SSNHL can occur in patients with COVID-19 regardless of infection severity,^5^^,^^34^ suggesting that the association between COVID-19 and SSNHL may be stronger than demonstrated in our study. Secondly, due to data restrictions, this study did not include audiological data such as pure tone thresholds or speech audiometry of the diagnosed patients. The lack of objective audiometric data and reliance on ICD-10 codes for HL and SSNHL diagnoses may lead to misclassification bias. Nevertheless, a study by Ko et al.^35^ demonstrated the feasibility of using administrative data with multiple diagnostic criteria to study SSNHL in a large population, supporting the validity of our approach. Moreover, South Korea has a strict process involving standardized testing methods for registering individuals with HL or SSNHL,^35^^,^^36^ which likely enhances the accuracy of diagnoses in this study. Thirdly, the generalizability of our findings to other populations requires caution, as they may not be applicable to different healthcare systems, genetic backgrounds, and virus variants. Fourthly, the retrospective study design, the higher proportion of participants with missing data for health screening examination results, and the lack of a longer follow-up to assess whether the risk of HL or SSNHL is modified over an extended period may also be limitations of this study. Lastly, this study did not specifically evaluate the effects of COVID-19 vaccinations against the risk of HL or SSNHL. While there is insufficient evidence regarding the association between COVID-19 vaccines and SSNHL, a large-scale cohort study has associated the BNT162b2 messenger RNA vaccine (Pfizer-BioNTech) with an increased risk of SSNHL,^37^ whereas another study reported no association among all types of COVID-19 vaccines.^38^ Although this study adjusted for the doses of COVID-19 vaccinations, further research fully considering the different types of vaccines is warranted.

In summary, our results suggest that COVID-19 may be an independent risk factor for HL and SSNHL in young adults with generally healthy otolaryngological conditions. It is recommended that even healthy young adults remain vigilant about the risk of HL and SSNHL following COVID-19 to enhance the prevention and management of COVID-19 sequelae. Further biological studies are warranted for a more comprehensive understanding.

## Contributors

YHO and MJS conceived and designed the study. All authors have accessed to and verified the underlying study data. HJK, SJ analysed the data. All authors contributed to data interpretation. HJK and SJ wrote the manuscript. KK, YHO and MJS revised the manuscript, and all authors approved the final version of the manuscript and had final responsibility for the decision to submit for publication.

## Data sharing statement

The data is available from the National Health Insurance Service (NHIS), which manages the data. Requests for access can be directed to NHIS through their website at http://www.nhiss.nhis.or.kr/.

## Declaration of interests

All authors declare no conflict of interests.
