# Supplementary material for: Incidence of hearing loss following COVID-19 among young adults in South Korea: a nationwide cohort study
Source: eClinicalMedicine. 2024 Jul 29;75:102759. doi: 10.1016/j.eclinm.2024.102759 (PMC11339059; doi:10.1016/j.eclinm.2024.102759)
Supplement: Supplementary Material [file mmc1.pdf]

Supplementary Table 1. Health screening examination participation and results of the young adults in South Korea

	Young adults (n=6,716,879)
Health screening examination	
Yes	3,336,346 (49.7)
No	3,380,533 (50.3)
Body mass index, kg/m^2, mean (SD)[a]	23.9 (4.3)
Systolic blood pressure, mmHg, mean (SD)[a]	117.4 (13.0)
Diastolic blood pressure, mmHg, mean (SD)[a]	72.9 (9.7)
Fasting serum glucose, mg/dL, mean (SD)[a]	93.4 (15.6)
Smoking status, n (%)	
Yes	980,269 (14.6)
No	2,355,727 (35.1)
Missing	3,380,883 (50.3)
Alcohol consumption, n (%)	
Yes	2,774,179 (41.3)
No	561,481 (8.4)
Missing	3,381,219 (50.3)
Moderate-to-vigorous physical activity, n (%)	
0	924,427 (13.8)
1-2	728,767 (10.8)
3-4	702,034 (10.5)
≥5	978,711 (14.6)
Missing	3,382,940 (50.4)

[a]Body mass index, blood pressure, and fasting serum glucose were missing for 3,382,640, 3,380,700, and 3,380,718 participants.
Acronyms: SD, standard deviation.

Supplementary Table 2. Subdistribution hazard ratios for the association of COVID-19 with the risk of hearing loss and sudden sensorineural hearing loss after multiple imputations for the covariates

	No COVID-19 (n=1,882,113)	COVID-19 (n=4,834,766)	*P* value
Hearing loss			
Number of imputations=1	1.00 (reference)	3.44 (3.33-3.56)	<0·0001
Number of imputations=2	1.00 (reference)	3.44 (3.36-3.53)	<0·0001
Number of imputations=3	1.00 (reference)	3.44 (3.38-3.51)	<0·0001
SSNHL			
Number of imputations=1	1.00 (reference)	3.52 (3.23-3.83)	<0·0001
Number of imputations=2	1.00 (reference)	3.52 (3.31-3.74)	<0·0001
Number of imputations=3	1.00 (reference)	3.52 (3.35-3.70)	<0·0001

aSHR (95% CI) calculated using the Fine-Gray subdistribution hazard regression model after adjustments for age, sex, household income, Charlson comorbidity index, COVID-19 vaccination, body mass index, hypertension, diabetes, dyslipidemia, smoking status, alcohol consumption, and moderate-to-vigorous physical activity.

Acronyms: COVID-19, coronavirus disease 2019; aSHR, adjusted subdistribution hazard ratio; CI, confidence interval; SSNHL, sudden sensorineural hearing loss.

Supplementary Table 3. Descriptive characteristics of the young adults in South Korea before and after the IPTW

	Before the IPTW		SMD	After the IPTW		SMD
	**No COVID-19 (n=1,882,113)**	**COVID-19 (n=4,834,766)**		**No COVID-19 (n=6,716,718)**	**COVID-19 (n=6,716,906)**	
Age, years, mean (SD)	29.7 (5.7)	29.4 (5.6)	-0.236	29.6 (6.7)	29.6 (10.5)	-0.005
Sex, n (%)			0.006			0.000
Male	929,965 (49.4)	2,361,422 (48.8)		3,294,319 (49.0)	3,291,867 (49.0)	
Female	952,148 (50.6)	2,473,344 (51.2)		3,422,399 (51.0)	3,425,038 (51.0)	
Household income, n (%)			-0.006			0.000
Upper half	964,062 (51.2)	2,446,221 (50.6)		3,408,969 (49.2)	3,410,111 (49.2)	
Lower half	918,051 (48.8)	2,388,545 (49.4)		3,307,748 (50.8)	3,306,795 (50.8)	
CCI, n (%)			0.045			0.000
0	735,721 (39.1)	1,672,074 (34.6)		2,408,158 (35.9)	2,407,877 (35.8)	
1	898,572 (47.7)	2,505,970 (51.8)		3,372,063 (50.2)	3,416,087 (50.9)	
≥2	247,820 (13.2)	656,722 (13.6)		936,497 (13.9)	892,942 (13.3)	
Hypertension, n (%)	50,276 (2.7)	131,402 (2.7)	-0.002	188,387 (2.8)	179,260 (2.7)	0.008
Diabetes, n (%)	17,081 (0.9)	42,684 (0.9)	0.003	65,144 (1.0)	57,843 (0.9)	0.011
Dyslipidemia, n (%)	48,061 (2.6)	119,936 (2.5)	0.005	181,684 (2.7)	177,857 (2.6)	0.004
COVID-19 vaccination, n (%)			-0.003			0.000
None	108,262 (5.8)	281,784 (5.8)		389,258 (5.8)	389,921 (5.8)	
Single dose	17,532 (0.9)	56,251 (1.2)		63,473 (0.9)	77,785 (1.2)	
Completion of primary series	1,756,319 (93.3)	4,496,731 (93.0)		6,263,986 (93.3)	6,249,200 (93.0)	

IPTW creates pseudo-population, where observations are weighted to represent a balanced population for both the case (i.e., COVID-19) and control (i.e., NO COVID-19) populations. The sample size of the pseudo-population had increased because of the size of the inverse probability of treatment weight.

Acronyms: IPTW, inverse probability of treatment weighting; SMD, standardized mean difference; COVID-19, coronavirus disease 2019; SD, standard deviation; CCI, Charlson comorbidity index.

Supplementary Table 4. Subdistribution hazard ratios for the association of COVID-19 with the risk of hearing loss and sudden sensorineural hearing loss after the inverse probability of treatment weighting

	**No COVID-19 (n=6,716,718)**	**COVID-19 (n=6,716,906)**	***P* value**
Hearing loss			
Event	13,831	47,604	
PMs	40,252,707	40,263,593	
Incidence/10,000 PMs	3.4	11.8	
SHR (95% CI)	1.00 (reference)	3.44 (3.38-3.51)	<0·0001
aSHR (95% CI)[a]	1.00 (reference)	3.44 (3.37-3.51)	<0·0001
aSHR (95% CI)[b]	1.00 (reference)	3.44 (3.38-3.51)	<0·0001
Sudden sensorineural hearing loss			
Event	2,092	7,366	
PMs	40,384,940	40,386,099	
Incidence/10,000 PMs	0.5	1.8	
SHR (95% CI)	1.00 (reference)	3.52 (3.35-3.70)	<0·0001
aSHR (95% CI)[a]	1.00 (reference)	3.52 (3.35-3.69)	<0·0001
aSHR (95% CI)[b]	1.00 (reference)	3.52 (3.36-3.70)	<0·0001

SHR calculated using the Fine-Gray subdistribution hazard regression model.

[a]adjusted for age and sex.

[b]adjusted for age, sex, household income, Charlson comorbidity index, COVID-19 vaccination, hypertension, diabetes, and dyslipidemia.

Acronyms: COVID-19, coronavirus disease 2019; PM, person-month; SHR, subdistribution hazard ratio; CI, confidence interval; aSHR, adjusted subdistribution hazard ratio.

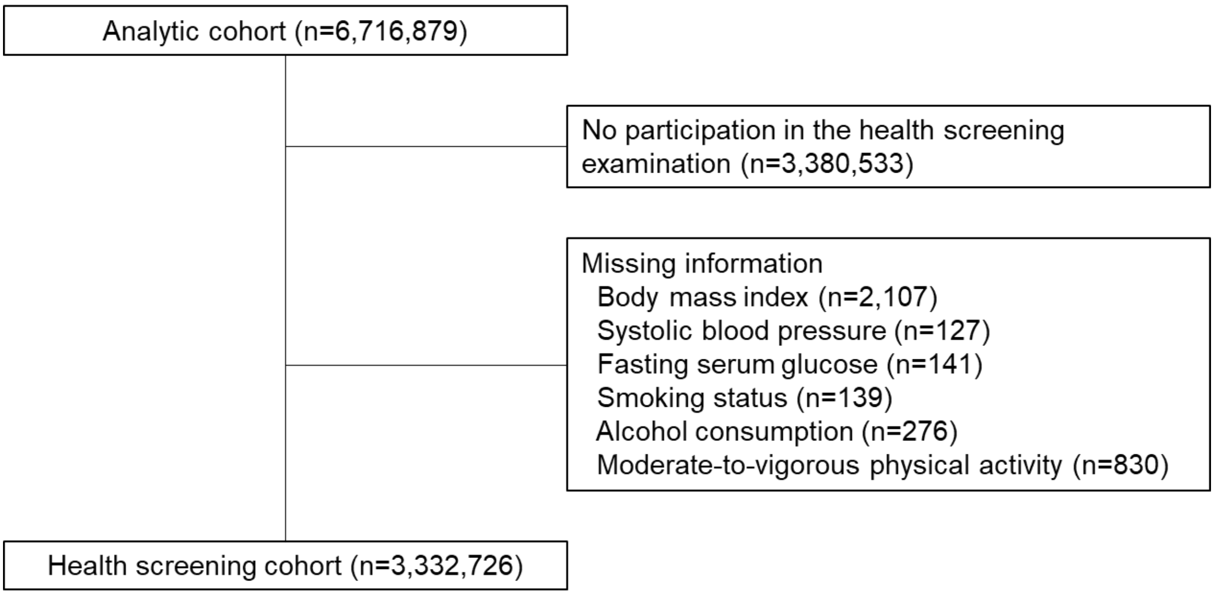

Supplementary Figure 1. Flow diagram for the inclusion of study participants in the health screening cohort

Supplementary Table 5. Descriptive characteristics of the young adults in South Korea who underwent health screening between 2020-2021

	**No COVID-19 (n=950,787)**	**COVID-19 (n=2,381,939)**
Age, years, mean (SD)	31.1 (4.9)	31.3 (5.0)
Sex, n (%)		
Male	466,016 (49.0)	1,151,369 (48.3)
Female	484,771 (51.0)	1,230,570 (51.7)
Household income, n (%)		
Upper half	518,203 (54.5)	1,290,292 (54.2)
Lower half	432,584 (45.5)	1,091,647 (45.8)
CCI, n (%)		
0	341,880 (36.0)	751,968 (31.6)
1	470,996 (49.5)	1,273,175 (53.5)
≥2	137,911 (14.5)	356,796 (15.0)
Hypertension, n (%)	29,316 (3.1)	75,087 (3.2)
Diabetes, n (%)	8,862 (0.9)	21,599 (0.9)
Dyslipidemia, n (%)	30,983 (3.3)	75,501 (3.2)
COVID-19 vaccination, n (%)		
None	39,812 (4.2)	105,530 (4.4)
Single dose	7,422 (0.8)	22,475 (75.2)
Completion of primary series	903,553 (95.0)	2,253,934 (94.6)
Body mass index, kg/m^2, mean (SD)	23.9 (4.3)	23.8 (4.2)
Waist circumference, cm, mean (SD)	78.8 (12.7)	78.6 (12.6)
Systolic blood pressure, mmHg, mean (SD)	117.4 (13.0)	117.3 (13.0)
Diastolic blood pressure, mmHg, mean (SD)	72.9 (9.7)	72.9 (9.7)
Fasting serum glucose, mg/dL, mean (SD)	93.4 (15.7)	93.4 (15.5)
Serum creatinine, mg/dL, mean (SD)	0.8 (0.3)	0.8 (0.3)
Smoking		
Yes	282,589 (29.7)	696,718 (29.3)
No	668,198 (70.3)	1,685,221 (70.7)
Alcohol consumption		
Yes	786,715 (82.7)	1,985,022 (83.3)
No	164,072 (17.3)	396,917 (16.7)
MVPA		
0	259,383 (27.3)	664,709 (27.9)
1-2	209,081 (22.0)	519,420 (21.8)
3-4	203,556 (21.4)	498,223 (20.9)
≥5	278,767 (29.3)	699,587 (29.4)

Acronyms: COVID-19, coronavirus disease 2019; SD, standard deviation; CCI, Charlson comorbidity index; MVPA, moderate-to-vigorous physical activity.

Supplementary Table 6. Subdistribution hazard ratios for the association of COVID-19 with the risk of hearing loss and sudden sensorineural hearing loss among young adults who underwent health screening

	No COVID-19 (n=950,787)	COVID-19 (n=2,381,939)	*P* value
Hearing loss			
Event	2,021	18,300	
PMs	5,697,255	14,274,289	
Incidence/10,000 PMs	3.5	12.8	
SHR (95% CI)	1.00 (reference)	3.61 (3.45-3.78)	<0·0001
aSHR (95% CI)[a]	1.00 (reference)	3.59 (3.43-3.76)	<0·0001
aSHR (95% CI)[b]	1.00 (reference)	3.55 (3.39-3.71)	<0·0001
aSHR (95% CI)[c]	1.00 (reference)	3.55 (3.39-3.72)	<0·0001
Sudden sensorineural hearing loss			
Event	329	2,875	
PMs	5,716,508	14,321,233	
Incidence/10,000 PMs	0.6	2.0	
SHR (95% CI)	1.00 (reference)	3.49 (3.11-3.91)	<0·0001
aSHR (95% CI)[a]	1.00 (reference)	3.46 (3.09-3.88)	<0·0001
aSHR (95% CI)[b]	1.00 (reference)	3.43 (3.06-3.84)	<0·0001
aSHR (95% CI)[c]	1.00 (reference)	3.43 (3.06-3.85)	<0·0001

SHR calculated using the Fine-Gray subdistribution hazard regression model.

[a]adjusted for age and sex.

[b]adjusted for age, sex, household income, Charlson comorbidity index, and COVID-19 vaccination.

[c]adjusted for age, sex, household income, Charlson comorbidity index, COVID-19 vaccination, body mass index, hypertension, diabetes, dyslipidemia, smoking status, alcohol consumption, and moderate-to-vigorous physical activity.

Acronyms: COVID-19, coronavirus disease 2019; PM, person-month; SHR, subdistribution hazard ratio; CI, confidence interval; aSHR, adjusted subdistribution hazard ratio.
